# Variations in Postnatal Care (PNC) Interventions Provided to Newborns in Health Facilities Across Kakamega County, Western Kenya

**DOI:** 10.24248/eahrj.v9i1.829

**Published:** 2025-09-30

**Authors:** Ruth Shitabule, Everlyne Morema, Tecla Sum, Morris Senghor Shisanya

**Affiliations:** a Department of Health, County Government of Kakamega; b Department of Community Health Nursing and Extension, School of Nursing, Midwifery and Paramedical Sciences (SONMAPS), Masinde Muliro University of Science and Technology (MMUST), Kakamega, Kenya; c Department of Paramedical Sciences, School of Nursing, Midwifery and Paramedical Sciences (SONMAPS), Masinde Muliro University of Science and Technology (MMUST), Kakamega, Kenya; d Department of Community Health Nursing, School of Nursing, Kibabii University, Bungoma, Kenya

## Abstract

**Introduction:**

Neonatal mortality remains high globally, with Kenya reporting a rate of 20 deaths per 1,000 live births. Essential Newborn Care (ENC) is crucial for preventing neonatal deaths, yet the provision of this care for the newborns is varied.

**Methods:**

This cross-sectional analytical study evaluated the provision of PNC-ENC across four scheduled visits in Kakamega County, Kenya. Data were collected from 325 mothers through structured questionnaires. Descriptive and inferential statistics, including ANOVA, were used to assess care provision.

**Results:**

The overall mean provision of expected newborn interventions across the four visits was 57.82%. Provision was highest at the first visit (59.55%), declined at 2 to 4 weeks (55.16%), improved at 4 to 6 weeks (59.06%), and slightly declined at 4 to 6 months (57.50%).

**Conclusions:**

This study highlights gaps in the delivery of essential newborn care, particularly in physical examinations. Targeted interventions, including training and resource allocation, are recommended to improve provision to PNC care interventions and reduce neonatal mortality.

## BACKGROUND

In the year 2022, 2.3 million children died in their first month of life globally translating to approximately 6,300 neonatal deaths each day.^1,2^ This is despite the significant reduction of up to 53% reported over the years since 1994. Neonatal mortality still remains high globally at 17 deaths per 1,000 live births in 2022 and even higher in Kenya at 20/100 live births.^3^ More than 75% of these newborn deaths occur within the first week of delivery. In Africa, 38% of infants’ deaths is associated with substandard Postnatal Care (PNC). The postnatal period within the continuum of maternal and newborn care is often overshadowed by the prenatal and delivery periods with possibility of inadequate care provision.^4^ This may frustrate efforts to achieve SDG 3 targets on maternal and neonatal mortality ratios by the year 2030.^5^

All newborns should have access to essential newborn care (ENC), which is the critical care required for all babies in the first days after birth.^6^ Essential newborn care involves immediate care at the time of birth, and essential care during the entire newborn period. It is needed both in the health facility and at home. In Kenya, health facility-based care is included in the Postnatal Care (PNC) package with specific interventions implemented in each of the 4 visits.^7^ World Health Organization recommends at least four postnatal contacts for all mothers and newborns within at 24 hours, 10 to 14 days, 4 to 6 weeks and 4 to 6 months after birth. In Kenya, the visits are scheduled; within 48 hours after birth, 1 to 2 weeks, 4 to 6 weeks, and 4 to 6 months. Following a home delivery, mother and newborn should be referred to the nearest health facility as soon as possible and preferably within 24 to 48 hours.^7^

Provision of ENC has been estimated at 23.51% in Africa in a systematic review but the variations across the 4 postnatal visits have not been documented.^8^ This rate of provision is extremely low and unfavourable considering the importance of this care for child survival. Providing optimal postnatal care (PNC) prevents both maternal and neonatal deaths, in addition to the prevention of long-term complications.^2,9^ Thus, there was a need to measure the provision of ENC in the current setting. This could provide insight for context specific strategies aimed at changing the status and contribute to the achievement of sustainable development goal 3. Further, understanding the variations across the 4 visits will provide guidance that will ensure optimum use of resources.

## METHODS

### Study Design and Setting

A cross-sectional analytical study was conducted in Kakamega County, Kenya. We adopted a convergent parallel methods design to compare visit-specific provision across Kenya's four scheduled PNC contacts. Kakamega is a populous county in the western region of Kenya with a total population of 1,867,579 people.^10^ Its health care system is comprised of several level one to four health facilities and one teaching and referral hospital (Kakamega County General Teaching & Referral Hospital). The county has different staffing patterns for the various cadres of health providers depending on the facility levels with medical doctors only at level four and five facilities.

### Study Population

The study population were newborns within 48 hours up to 6 months of age whose mothers sought postnatal care (PNC) at the postnatal ward and MCH clinics in the selected facilities. The respondents were the postnatal mothers and the midwives working in these service areas. Quantitative data was collected using an interviewer administered questionnaire/checklist (mothers) and self-administered questionnaires (midwives).

### Sample Size and Sampling Technique

The study employed multistage sampling. Facilities were first clustered according to levels. High volumes facilities were selected from each level (2, 3, 4 & 5). The sample size of n=325 of newborns, derived using Fisher's formula with p=.50, Z=1.96, margin d=0.055, design effect=1.0, and ~2% allowance for non-response yielded n≈325, was proportionately distributed per facility based on the number of deliveries in a month. All mothers with newborn babies who consented (n=325) and were in the service provision areas in the various facilities during the data collection month responded to the mothers’ interviewer administered questionnaire with regard to the care instituted by health care providers for their babies. Furthermore, self-administered questionnaires were given to n=160 midwives (derived by Fisher's formula and corrected for finite population) who were on duty when mothers from each facility and gave consent were interviewed to correlate the findings. Midwives (n=160) were sampled from the duty-roster frame with finite population correction (N=273 eligible midwives]); this provider strand was not pair-matched to mother–baby interviews.

### Data Collection and Data Collection Tools

The two questionnaires and checklists underwent content validation by subject experts and pilot-testing in non-study facilities (n=30) to assess clarity, flow, and timing; minor wording and ordering changes were made; median completion time was 22 minutes. To minimize recall bias interviews were anchored to the index child's PNC schedule and verified against MCH booklets/registers when available; recall was limited to ≤6 months postpartum; show-cards and neutral probes were used. Both the postnatal mother and midwives’ questionnaires were administered on m-Health platform tool (Kobo collect) and the data exported to IBM SPSS Statistics for Windows version 25.0 (IBM Corp, Armonk, NY), for analysis. Data was collected between January and March 2024.

### Variables and Variable Measurements

#### Primary Outcome (visit-specific provision of newborn PNC)

The primary outcome is the proportion of expected newborn postnatal care (PNC) interventions provided at each scheduled contact (within 48 hours, 1–2 weeks, 4–6 weeks, and 4–6 months). For each visit, mothers were asked whether specific, guideline-aligned interventions were provided during that contact. Items included (but were not limited to): thermal care, counseling, exclusive breastfeeding counseling/assessment, cord care counseling, danger-sign assessment, physical examination components (e.g., weight, temperature, jaundice/skin, umbilicus, respiratory assessment, feeding check), growth monitoring, and immunization (at age-appropriate visits). Each item was coded Yes = 1 / No= 0. For each visit, a visit-specific composite score was calculated as the mean of available items × 100, yielding a percentage (0–100%). Items not applicable to a given visit (e.g., vaccinations at ≤48 h) were excluded from that visit's denominator. Higher scores indicate more complete provision of newborn care at that contact.

#### Secondary Outcomes

Overall provision (all visits combined): The mean of the four visit-specific composites expressed as a percentage and domain-specific provision like clinical examination domain: Mean of binary items reflecting hands-on examination (e.g., weight, temperature, respiratory check, skin/jaundice, cord, feeding check). Finally, Item-level coverage: Proportion (percent) of newborns receiving each individual intervention per visit.

### Data Analysis

Descriptive statistics were used to summarise the respondents’ sociodemographic characteristics. We assessed normality (Shapiro–Wilk) and homogeneity (Levene). Where violated, we used Welch ANOVA with Games–Howell post-hoc; conclusions were robust. Item-level missingness was summarised; primary analyses used listwise deletion, with pairwise deletion as sensitivity analyses (similar estimates). A one-way ANOVA with pairwise comparison was used to determine whether the differences in the mean proportion of provided care interventions between the visits were significant. Significance was set at *p*≤.05 with a 95% confidence interval.

### Ethical Considerations

Ethical considerations were duly followed; clearance was sought from Institutional Ethical Review Committee of Masinde Muliro University of Science and Technology. The research permit was obtained from the National Commission for Science, Technology and Innovations (NACOSTI) as required by law in Kenya. Permission to conduct the study was sought from Kakamega county referral and teaching hospital and the other selected facilities within the county. Informed consent was sought and comprehension affirmed from the respondents.

## RESULTS

**FIGURE 1:**
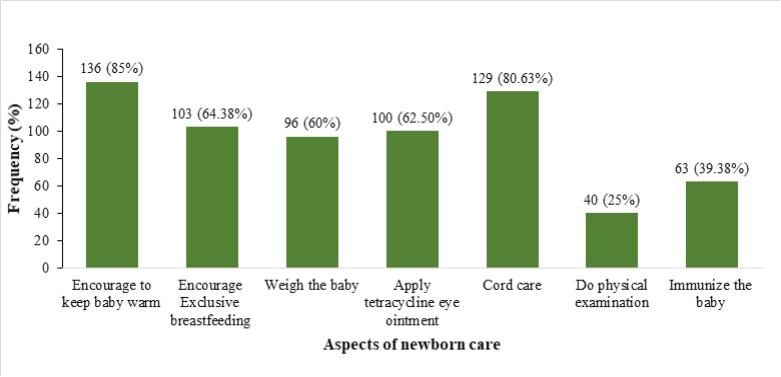
Immediate Newborn Care (Within 48 Hours)

The mean proportion of expected intervention across the different visits was estimated at 57.82% The mean proportion for the first visit was 59.55%, with a standard deviation of ±20.09. A good number of mothers (85%) reported to have been encouraged to keep the baby warm to minimise loss of body heat thus preventing hypothermia. Similarly, approximately 64.38% of nurses advocated for exclusive breastfeeding, promoting optimal nutrition and bonding between mother and baby. At least 60% of the newborns were reported to have been weighed in postnatal visit. Unfortunately, only 25% of newborns were reported to have undergone a physical examination, which typically includes assessing vital signs, reflexes, and overall health status. This is further described in Figure 1.

**FIGURE 2:**
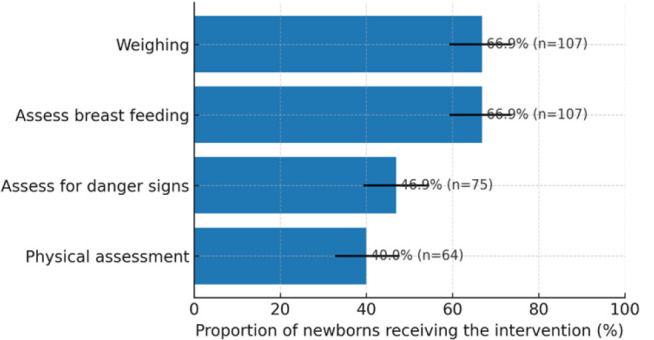
Care to the Newborn for 1–2 Weeks Postnatal Natal Care visit (n=160)

A second postnatal visit is usually made between 2 to 4 weeks with a number of interventions expected to be provided for the newborns. A mean proportion of 55.16% of the expected interventions were provided during the visit with a standard deviation of ±20.81. Physical examination was conducted for 40% of the newborns were found to have been physically examined. This is further summarized in figure 2.

### Third Postnatal Care Visit (4 to 6 Weeks Post-Delivery)

**FIGURE 3:**
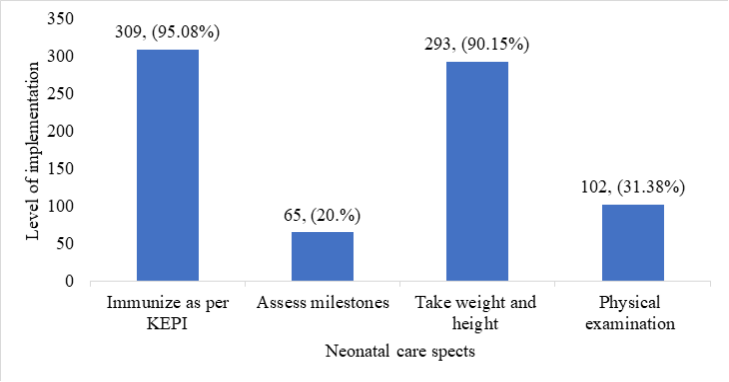
Interventions on the 3rd Postnatal Care Visit

The third postnatal visit for both the mother and the baby is made between the 4^th^ and 6^th^ weeks post-delivery. The mean percentage level of care provided to newborns during this visit was estimated to be 59.06% with a standard deviation of ±15.73. At least 31.25% of the newborns were reported to have been examined physically, while weight and length measurements were taken for 90% of the newborns. Further, 95% of the newborns received immunisations as per the KEPI schedule. This is summarised in the chart (Figure 3).

### Fourth Prenatal Care visit

The fourth visit done between 4 to 6 months is usually the last postnatal visit for both mother and baby. Only the babies continue with immunisation and growth monitoring six monthly until they attain 5 years of age. During this visit mean percentage of care was 57.50%, with a standard deviation of ±19.79. At least 95.63% of newborns were immunised and 88.13% had their weight and height measurements taken. Additionally, complete physical examinations and assessment of danger signs were performed by 24.38% and 21.88% of nurses, respectively. (Table 1)

**TABLE 1:** Care for the Newborn at 4 to 6 Months Postnatal Care Visit (N=325)

Intervention	N	Percentage (%)	95% CI (Lower)	95% CI (Upper)
Immunisation	310	95.38	92.53	97.18
Weight/height	286	88.0	4.02	91.1
Physical exam	79	24.31	19.96	29.25
Danger-sign assessment	71	21.85	17.7	26.65

Key to abbreviations: CI, confidence interval

### Variations of the Newborn Care Provision Across the Different Postnatal Visits

**FIGURE 4:**
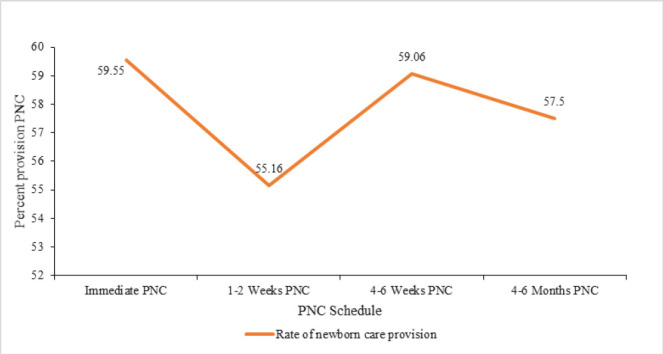
A Line graph depicting the variations in provision of care interventions across the four visits

Provision of newborn care exhibits fluctuations across the scheduled visits, with highest rate being observed at the first PNC visit within 48 hours of birth at 59.55%. This is followed by a slight decrease at 2–4 weeks PNC (55.16%), a subsequent increase at 4 to 6 weeks PNC (59.06%), and a slight decrease again at 4 to 6 months PNC (57.5%). (Figure 4)

The analysis indicates significant variations across different postnatal visits. The ANOVA results in Table 2 show a pairwise comparisons of a mean provision of newborn care during different postnatal periods. There was a significant mean difference (MD) in the proportions of care interventions provided between the immediate postnatal visit (within 48 hours of birth) and the 2 to 4 weeks postnatal care visit. However, no significant differences were observed between the immediate postnatal visit and in either the 4 to 6 weeks PNC) or the 4 to 6 months PNC. At the 2 to 4 weeks PNC visit, a significant mean difference in provision was observed when compared with the 4 to 6 weeks PNC visit. No significant difference was observed between provision at the 2 to 4 weeks PNC visit and the 4 to 6 months PNC visit. Similarly, no significant difference is found between provision at the 4 to 6 weeks PNC visit and the 4 to 6 months PNC visit.

**TABLE 2:** Pairwise Comparisons of Provision of Newborn Care Interventions During Different Postnatal Periods

Newborn Care (A)	Newborn Care (B)	Mean Difference (A-B)	95% CI	P Value
Immediately after birth	1–2 weeks PNC	4.40	1.24–7.55	.007
	4–6 weeks PNC	0.49	−2.35–3.33	.733
	4–6 months PNC	2.05	−0.62–4.72	.131
1–2 weeks PNC	4–6 weeks PNC	−3.91	−6.83–−0.98	.009
	4–6 months PNC	−2.34	−5.34–0.65	.124
4–6 weeks PNC	4–6 months PNC	1.56	−1.02–4.14	.233

Notes: ANOVA for Pairwise comparison based on estimated marginal means. Mean provision of the newborn care immediately after birth =59.55; 1–2 weeks PNC=55.16; 4–6 weeks PNC=59.06; and 4–6-months PNC=57.50. *P*<.05.

## DISCUSSION

Despite global efforts to reduce neonatal mortality, Kenya continues to experience high neonatal mortality rates, currently standing at 20 deaths per 1,000 live births in 2022.^3^ The World Health Organization (WHO) and other bodies advocate for essential newborn care (ENC) as a vital intervention to reduce these deaths. This study analysed the variations in postnatal care (PNC) interventions provided to newborns in health facilities across Kakamega County, Western Kenya. These interventions included; immediate care at birth, exclusive breastfeeding, thermoregulation, cord care, and timely immunisations, which are all essential for ensuring newborn survival.^7^ However, the study's findings reveal gaps in the provision of essential newborn care practices, with notable fluctuations in provision across the four scheduled PNC visits, similar to trends observed in other sub-Saharan African countries.^1,2,8,9,11–13^

The rate of provision of these interventions was found to be moderate in this study. This aligns with other researches done in low- and middle-income countries (LMICs), where inadequate healthcare infrastructure, staff shortages, and limited training impede the consistent delivery of postnatal services. A cross-sectional study based on DHS data from 23 low-income and middle-income countries (LMICs) across all the regions estimated PNC care provision at 41.42%.^14^ A pooled magnitude of postnatal care utilization in sub-Saharan African countries was 52.48% in a metanalysis of DHS data among 36 sub-Saharan Africa countries.^13^ A cross-sectional study from northern Ethiopia reported it at 45.1%. Yet, rates as low as 23. 51% were reported by a secondary analysis of similar data from 21 sub-Saharan African countries that specifically focused on the care for the newborn.^1^ This discrepancy implies an imbalance in care provision between the mother and the newborn during this period.

The first postnatal visit had the highest number of infants receiving most of the interventions. Most mothers were encouraged to keep their newborns warm, received cord care, and a significant number had early exclusive breastfeeding initiation. This is slightly higher that the rate reported from Ethiopia for this period.^15^ This difference can be accounted for by the passage of time with possible improvement of strategies since the latter rate was based on data from demographic surveys conducted up to the year 2021. This agrees with the observation that women who deliver in health facilities are likely to receive prompt PNC alongside their children as long as the mother does not develop complications.^1^ Early postnatal care is critical, as 75% of neonatal deaths occur in the first week of life, primarily due to preventable causes such as infections, hypothermia, and complications from preterm birth. However, only 25% of newborns received a physical examination. A study in Uganda similarly reported low rates of postnatal physical assessments, with only 30% of newborns being examined within 24 hours.^16^

On the second postnatal visit at 1 to 2 weeks, care provision dropped to 55.16%, with only 40% of newborns undergoing physical examinations. Inadequate follow-up care during this period is a common challenge in resource-limited settings. Research from Tanzania showed that only 14.6% of newborns returned for the second PNC visit, largely due to logistical barriers such as distance to health facilities, not being scheduled for the visit and cultural factors.^17^ The failure to maintain high rates of provision during this visit is alarming, given that complications from infections and feeding issues can manifest within the first week after birth.^6^

The third postnatal visit is expected between 4 to 6 weeks post-delivery. During this visit, the general condition of the baby should be assessed, a complete physical examination should be performed, weight and length of the baby measured and recorded in the growth chart, baby should be assessed for developmental milestones (smile, eye movement) and given immunization (second dose of OPV and first dose of the Pentavalent (DPT/HEB/HIB) vaccine).^18^ In this study, the rate of newborn care intervention improved slightly from the previous visit to 59.06%, with 90% of newborns being weighed and measured, but only 31.25% receiving a physical examination. In this visit, there were higher rates of immunization at 95.63% which implies that focus for these visits in this setting could have been immunization services. Meanwhile, physical examination rates remained as low as 24.38%. These figures suggest that while basic interventions like immunizations and weight measurements are prioritized, comprehensive examinations are often neglected. Studies from Nigeria and Ethiopia have highlighted similar gaps in care, where critical assessments, such as milestone tracking and danger sign evaluations, are frequently overlooked due to staffing constraints and time pressures.^8,11^

The last targeted postnatal visit is usually expected between 4 to 6 months with the expectations that all infants will be assessed of immunisation status, weighed, completely physically examined and assessed for baby danger signs.^7^ In this study a mean of 57.5% of the infants received the aforementioned interventions. About 96% of the infants were immunized while, a paltry, 22% were observed for danger signs. This is expected considering that the baby has made considerable growth and has bonded with the mother to a level that danger signs can easily be decerned. Furthermore, there was scanty literature reporting on this particular visit.

There were significant gaps across the four visits in the provision of the PNC newborn care. The most substantial gap was between the immediate postnatal period and the second visit at 1 to 2 weeks which is a decline in the care. A significant increase is noted between the second visit and third visit. The Kenya postnatal care guidelines advocate for a home visit at 1 to 2 weeks by a community health promoter. Consideration could be made for follow up care by midwives at home based on the concept of community midwifery. However, no significant differences were found between the immediate postnatal period and the third and fourth visits, suggesting that care delivery stabilizes at suboptimal levels after the second visit. These results are consistent with findings from other LMICs, where health system limitations and socio-economic barriers impede continuous care delivery.

### Limitations of the Study

This cross-sectional, facility-based study cannot infer causality and may be subject to selection bias. Some measures relied on maternal self-report without universal objective verification; findings may not generalize to non-facility births. Potential clustering by facility and item-level missingness are acknowledged; sensitivity checks yielded similar results. However, this study provide insight into the variations and gaps in the care provided across the four postnatal visits that have not been previously documented.

## CONCLUSION

The results of this study underscore the need for targeted interventions to improve provision of newborn care, particularly physical examinations and danger sign assessments. Strengthening the capacity of healthcare providers especially community health promotors through training, improved supervision, and resource allocation is critical in enhancing care delivery for the second postnatal visit at week. Additionally, innovative approaches such as mobile health (mHealth) technologies, which have been shown to improve postnatal care follow-up rates in settings like Malawi and India, should be considered for use in Kakamega County.^9^ Collaboration between government agencies, healthcare institutions, and community health workers is necessary to ensure comprehensive newborn care and to achieve Sustainable Development Goal 3, which aims to reduce neonatal mortality to at least 12 deaths per 1,000 live births by 2030.^5^
